# Supplementary figures and images for: Genomic and Resistance Epidemiology of Gram-Negative Bacteria in Africa: a Systematic Review and Phylogenomic Analyses from a One Health Perspective
Source: mSystems. 2020 Nov 24;5(6):e00897-20. doi: 10.1128/mSystems.00897-20 (PMC7687029; doi:10.1128/mSystems.00897-20)

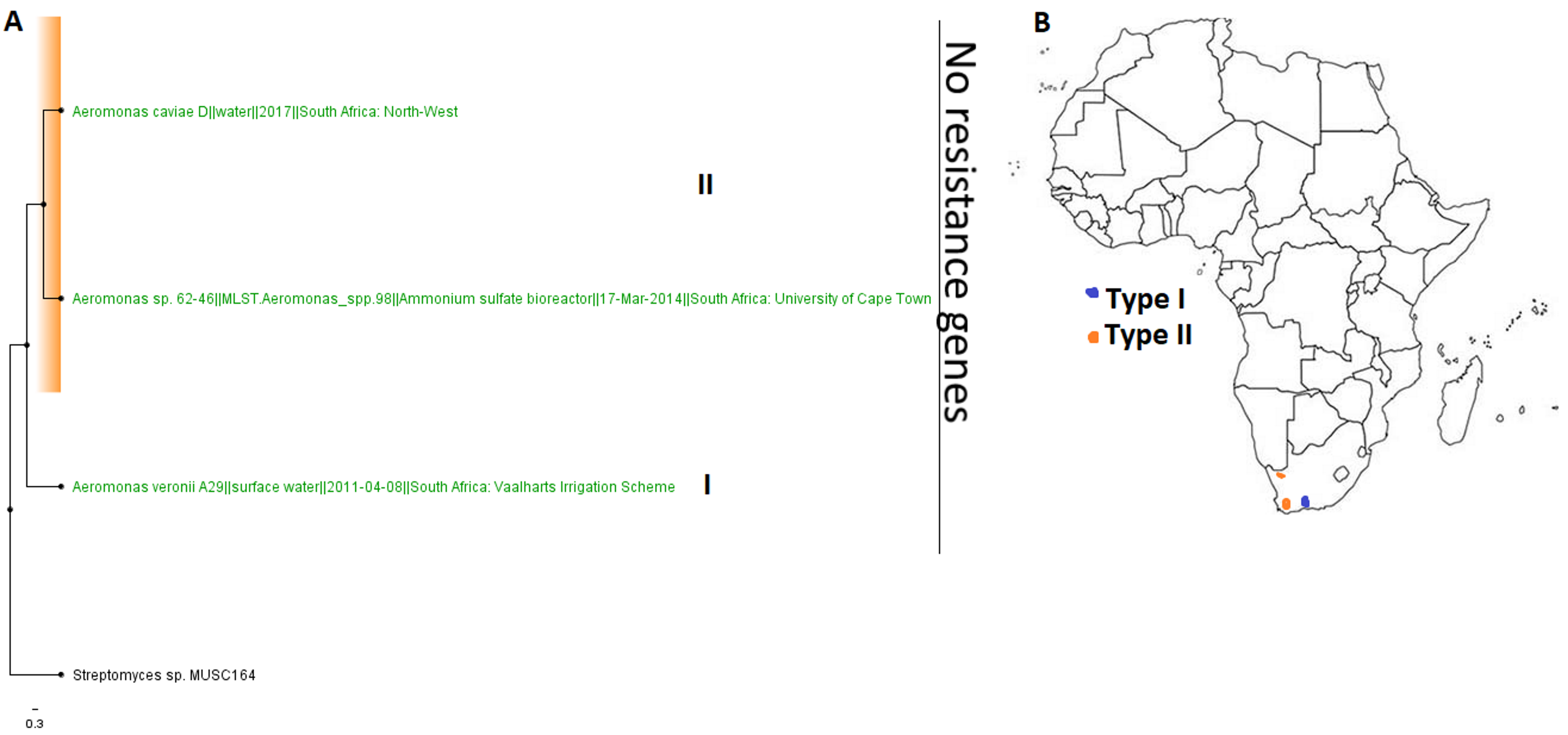

Supplement: FIG S1 [file mSystems.00897-20-sf001.tif]

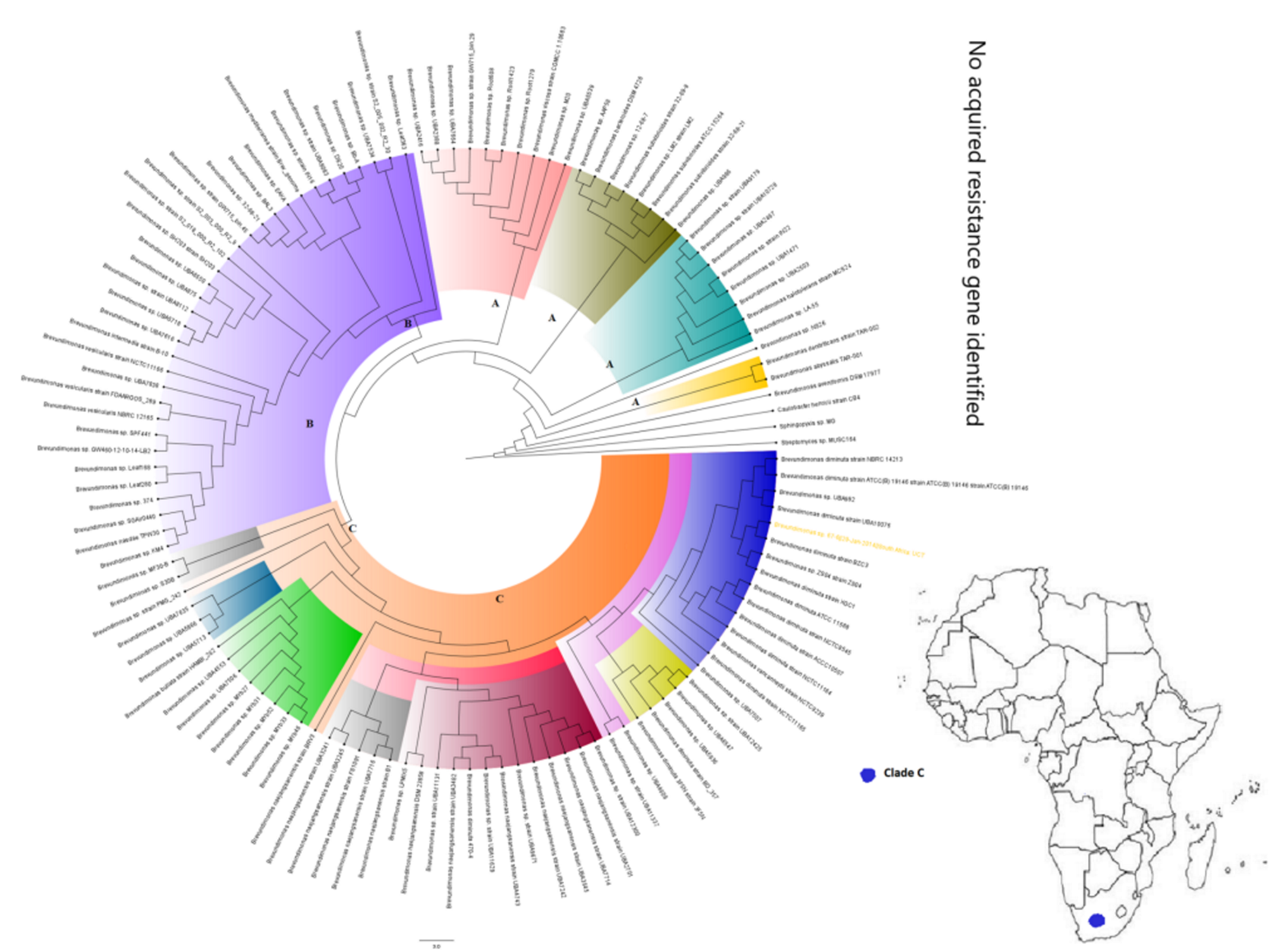

Supplement: FIG S2 [file mSystems.00897-20-sf002.tif]

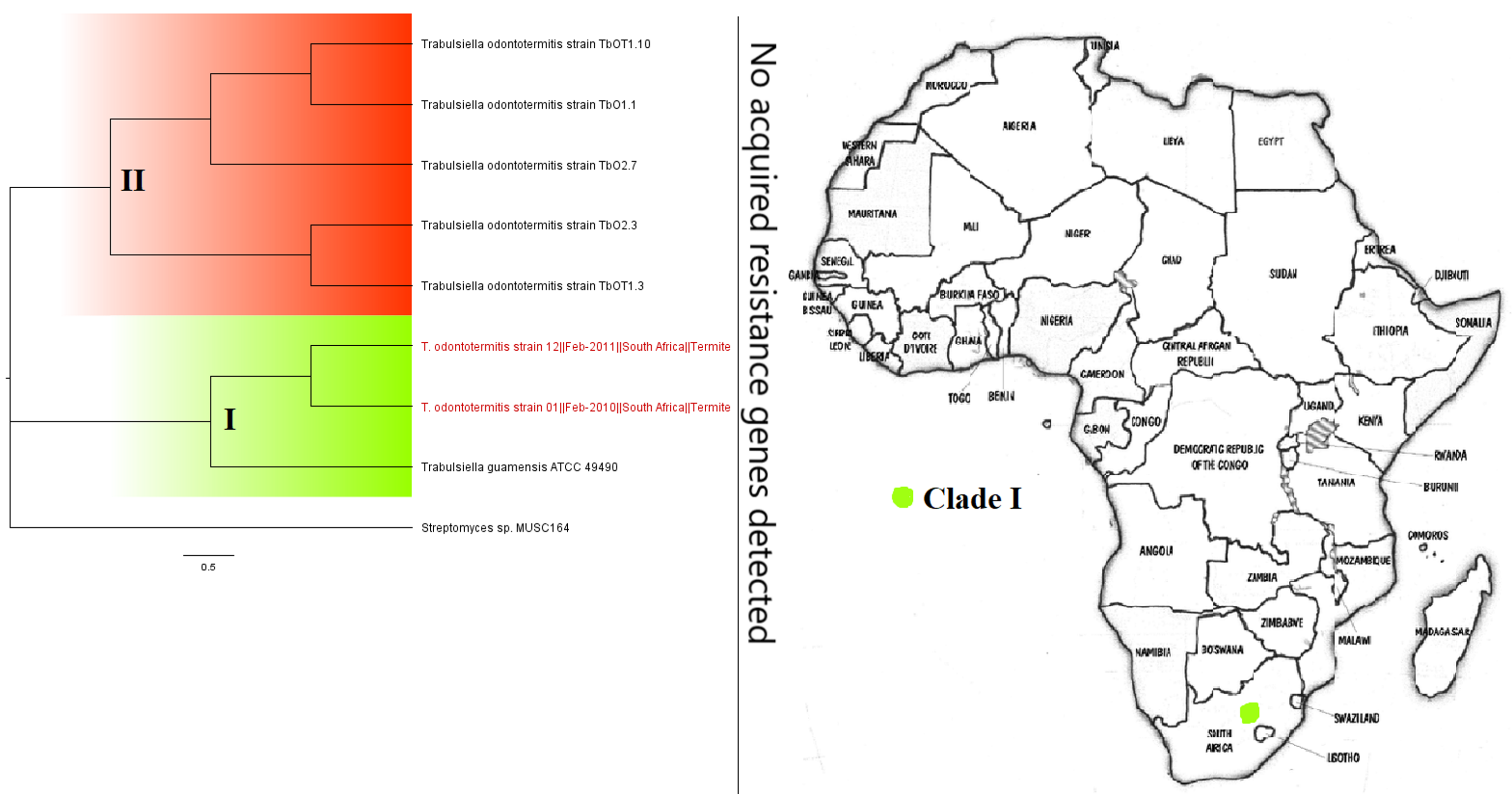

Supplement: FIG S3 [file mSystems.00897-20-sf003.tif]
